# Risk factors for cytomegalovirus disease in seropositive renal transplant recipients; a single-center case-controlled study

**DOI:** 10.15171/jnp.2017.39

**Published:** 2017-04-02

**Authors:** Viviana Navarro-Rodríguez, Alvaro Herrera-Munoz, Adrián Castro, Allan Ramos-Esquivel

**Affiliations:** ^1^Nephrology Department, Hospital San Juan de Dios, San José, Costa Rica; ^2^Department of Pharmacology, University of Costa Rica, San José, Costa Rica

**Keywords:** Cytomegalovirus, Kidney transplantation, Mycophenolate mofetil, Thymoglobulin, Transplant

## Abstract

**Background:**

Risk factors for cytomegalovirus (CMV) disease in renal transplant recipients have been evaluated in industrialized countries with relatively low CMV seroprevalence.

**Objectives:**

We aimed to determine which factors are related to this illness in a high CMV seroprevalence country.

**Patients and Methods:**

A case-control study was performed with data from a 5-year follow-up of 260 kidney transplant recipients at our center. Odds ratios were calculated using the Mantel-Haenszel method.

**Results:**

A total of 25 cases of CMV disease occurred during the study period. Recipient age greater than 55 years old (odds ratio: 4.95, 95% CI: 1.44–17.0) and use of thymoglobulin (odds ratio: 4.84; 95% CI: 1.10–21.39) were the only independent predictors for CMV disease. There was not any relationship between the previous serologic status of both donor and receptor and the occurrence of CMV disease. We did not observe any association between the immunosuppressive regimens and CMV disease, except for thymoglobulin.

**Conclusions:**

Only recipient age and thymoglobulin administration were related to CMV disease. Further studies are needed to determine if prophylactic treatment confers clinical benefit in this subset of patients.

**Implication for health policy/practice/research/medical education:**

Cytomegalovirus (CMV) disease in Costa Rica as a high seroprevalence country is related to recipient’s age and thymoglobulin administration.

## 1. Background

Cytomegalovirus (CMV) is the most important viral pathogen found after kidney transplant and is one of the main causes of morbidity from an infective source. A 20 to 60% of symptomatic infection incidence has been described among recipients of kidney allograft that did not receive prophylaxis or pre-emptive treatment (1).CMV disease has been associated with acute rejection, chronic allograft nephropathy, graft loss, cardiovascular disease, opportunistic infections and overall mortality (2-5).

Several risk factors for CMV disease in kidney recipients have been clearly identified in different series. Aging, comorbidities, immunosuppressive treatment at high doses and the serological status of both donor and receptor, being donor positive and receptor negative the most risky situation, have been all related to higher odds of CMV disease (6,7).

Nevertheless, the majority of these studies have been carried out in industrialized countries with relatively low CMV seroprevalence. In contrast, the seroprevalence of CMV in some developing countries such as Costa Rica is greater than 95% in adults representing a completely different clinical scenario (8).

## 2. Objectives

We undertook a case-control study in order to determine potential risk factors for our population and to establish if any immunosuppressive combination had an impact on the occurrence of CMV disease.

## 3. Patients and Methods

### 3.1. Study population

A case-control study was performed with data from consecutive patients from the nephrology department from the San Juan de Dios Hospital in San José, Costa Rica. An annual average of fifty renal transplants is performed in our center. Receptors of kidney transplant with CMV disease were identified during the period of time from January 2010 through December 2014. All patients were under immunosuppressive treatment with calcineurin inhibitors (tacrolimus or cyclosporine), prednisone and mycophenolate or azathioprine. Only patients receiving thymoglobulin were considered for treatment (n = 9).

### 3.2. Case definition

A case was defined as a patient with renal transplant who presented with CMV disease according to the American transplant association definition (9); a recipient with evidence of infection with CMV (circulating CMV antigenemia or PCR of viral DNA) and attributable symptoms.

### 3.3. Definition and selection of controls

For every case, two controls were selected randomly out of the pool of patients with renal transplant who did not have clinical evidence of CMV disease. They received the transplant in the same period as controls did and they also received comparable immunosuppressive drugs. They also had a similar follow-up time after transplantation. We did not include patients with HIV infection.

### 3.4. Data collection

The following data was retrospectively collected from medical records: age, gender, type of donor (living related or post-mortal), associated comorbidities (diabetes or high blood pressure), immunosuppressive treatment [prednisone, mycophenolate mofetil/ azathioprine, tacrolimus/cyclosporine, anti-thymocyte globulin (ATG)], and CMV serologic status of both donor and receptor (IgM/IgG). We also recorded whether a patient received CMV prophylaxis or not. Routine follow-up to our patients consisted of monthly clinical examination with evaluation of serum creatinine, urine protein and complete blood count. All patients with symptoms or signs suggestive of CMV disease (according to the nephrologist on charge) were required to have circulating CMV antigenemia or polymerase chain reaction (PCR) of viral DNA.

The time period between the renal transplant and the onset of CMV disease was calculated according to our registries. For patients with CMV disease, drugs taken at the time of diagnosis were determined. For controls all treatments used at the same period of time were included.

All acute graft rejections that took place between the transplant and the diagnosis were considered for the analysis, as well as the co-infections presented during the follow-up period.

Viral titers were acquired at time of diagnosis. They were determined by quantification of viral titer for *DNA* CMV by real-time PCR technique (cytomegalovirus PCR kit; Abbott Diagnostics, France) and performed at the Molecular Biology Laboratory at our center according to the manufacturer´s instructions.

The serologic determination for CMV previous to the transplant was performed by *ELISA* technique (Architech, Abbot, USA)

### 3.5. Ethical issues

The study was approved by the Local Bioethics Institutional Committee (CLOBI-HSJD-034-2013). The research followed the tenets of the Declaration of Helsinki; informed consent was obtained; and the research was approved by the ethical committee of Hospital San Juan de Dios, San José, Costa Rica.

### 3.6. Statistical analysis

Data are presented as median ± standard deviation (for quantitative variables) or as percentages (for qualitative variables). The comparison of clinical features for cases and controls was performed by student *t* test for independent samples (for continuous variables) or using the Chi-square test or Fisher’s exact test when applicable (for categorical variables). Odds ratios were calculated using the Mantel-Haenszel method in order to identify potential risk factors for CMV disease. Multivariate analysis was performed using stepwise logistic regression. Variables were introduced into the model if they had a *P* value less than 0.10 in the univariate analysis. Every p value had two tails. A *p* value less than 0.05 was considered statistically significant. The statistical analysis was performed by SPSS program for Mac 20.0 (Chicago IL. USA).

## 4. Results

During the study period 260 patients received a kidney transplant in our center. Mean follow-up time was 73 months. We identified 25 cases of CMV disease for a cumulative incidence of 9.62% per year. Table 1 summarizes demographic and clinical data from our patients and shows univariate comparisons between cases and controls for each studied variable. Only recipient’s age was significantly different between cases (46.7 ± 13.1 years versus controls with ages of 39.1 ± 13.8 years; *P *= 0.02). A total of 17 cases (68%) developed CMV disease in the first trimester after transplantation, one case between the 3 and 6 months period after surgery and 7 cases (28%) developed their disease 6 months or later after transplantation. The most frequent manifestations of CMV disease were gastrointestinal symptoms (diarrhea, abdominal pain, nausea and vomiting) that occurred in 9 cases (36%), followed by decreased in creatinine clearance in 5 cases (20%), respiratory symptoms in 4 cases (16%) and pancytopenia in only one case. Constitutional symptoms were exhibited in 5 cases (20%).

**Table 1:** Univariate analysis of potential risk factors for CMV disease

**Variable**	**Cases** **(n = 25)**	**Controls** **(n = 50)**	**Odds ratio** **(95% CI)**	**P value**
Recipient age > 55 years (%)	9 (36)	5 (10)	4.95 (1.44–17.0)	0.01
Male gender (%)	15 (60)	32 (64)	0.87 (0.32–2.34)	0.73
Life donor (%)	7 (28)	16 (32)	0.80 (0.28–2.31)	0.72
Diabetes mellitus (%)	3 (12)	12 (24)	0.47 (0.12–1.88)	0.22
High blood pressure (%)	18 (72)	36 (72)	1.03 (0.35–3.01)	0.99
Immunosuppressive Treatment (%)				
Prednisone	25 (100)	50 (100)	NA	0.69
Mycophenolate mofetil	20 (80)	41 (82)	0.78 (0.23–2.69)	0.21
Azathioprine	0 (0)	3 (6)	NA	0.81
Cyclosporine	5 (20)	11 (22)	0.86 (0.26–2.83)	0.54
Tacrolimus	20 (80)	36 (72)	1.44 (0.45–4.64)	0.47
Serologic status (%)				
D+/R+	23 (92)	50 (100)	1.07 (0.94–1.23)	0.12
D+/R-	0	0	NA	NA
D-/R+	2 (8)	0	1.06 (0.94–1.20)	0.30
Retransplant (%)	2 (8)	2 (4)	2.04 (0.27–15.43)	0.48
Prophylaxis (%)	3 (12)	6 (12)	1.20 (0.26–5.49)	0.80
Acute rejection (%)	8 (32)	8 (16)	2.41 (0.78–7.48)	0.11
Chronic rejection (%)	0	1 (2)	0.98 (0.94–1.02)	0.47

D: donor; R: recipient.

Table 2 presents the univariate analysis of potential risk factors for developing CMV disease according to immunosuppressive treatment received. None of the immunosuppressive regimens were associated with the risk of having CMV disease. Only patients who received thymoglobulin exhibited a significant increased risk of CMV disease (Odds ratio: 4.84, 95% CI: 1.10 – 21.39).

**Table 2:** Univariate analysis of immunosuppressive regimens and the risk of CMV disease

**Treatment**	**Cases** **No. (%)**	**Controls** **No. (%)**	**Odds ratio**	**95% CI**	**P value**
MMF + TAC	16 (64)	31 (62)	1.03	(0.38 – 2.81)	0.95
MMF + CSA	5 (20)	10 (20)	0.98	(0.29-3.29)	0.96
Thymoglobulin	6 (24)	3 (6)	4.84	(1.10-21.39)	0.03
MMF > 1500 mg/d	5 (25)	18 (36)	0.43	(0.13-1.39)	0.15
PDN > 15 mg/d	10 (40)	19 (38)	1.05	(0.39-2.82)	0.92

Abbreviations: CSA: cyclosporine; MMF: mycrophenolate mofetil; TAC: tacrolimus.

There was a significant association between pneumocystis (*P *= 0.01), pulmonary aspergillosis (*P *= .03), and CMV disease (12% and 16% of cases versus 0% for controls, respectively)

In the multivariate analysis, the use of thymoglobulin and recipient age greater than 55 years were both independently associated with the risk of CMV disease. (Odds ratio for thymoglobulin: 5.32, 95% CI: 1.13 – 25.03; Odds ratio for recipient age > 55 years: 5.29, 95% CI: 1.48–18.92).

## 5. Discussion

We conducted a retrospective review of our database through 5 years of follow-up in order to identify potential variables associated with the risk of developing CMV disease.

Our cumulative incidence (9.62 % per year) is very low in comparison to the rates reported by previous authors, ranging from 25% (10,11) to 60% (1). These contradictory results can be the consequence of different prophylactic measures in each study, as well as diverse techniques for the detection of CMV. Nevertheless, we consider that another source for such variation is the low proportion of patients with the D+/R- serostatus in our study, which is one of the most consistent risk factors for CMV disease. Moreover, it can also be the result of few patients receiving anti-lymphocyte therapy for immunosuppression, another well-known predisposing factor for this disease (12,13).

It has been described a high incidence of CMV disease during the first three months after surgery among those patients who did not receive prophylaxis and among those who were under high doses of immunosuppressive treatment (1). In concordance with this and previous studies (13), the majority of our cases (68%) presented their illness during the first trimester after transplantation, probably due to a major immunosuppressive state during this period of time (14). Furthermore, the clinical picture of patients with CMV disease is very similar to that reported by previous authors (1).

In this study, we showed that recipient’s age was significantly associated to CMV disease. Specifically, recipient’s age greater than 55 years was an independent risk factor for developing symptoms attributable to CMV. This relationship has been well documented previously and it seems to be related to a high CMV seroconversion rate in older people (4). However, the elevated CMV seroprevalence found in our study does not support this hypothesis. On the contrary, new findings have shown that latent CMV infection accelerates age-related changes in the T cell subsets in elderly patients, leading to a reduced proportion of naïve and early memory T cells and an increased number of CD8+ effector T cells which produce gamma interferon but do not have enough growth potential, making CMV disease more probable (15,16). Considering the previous finding, CMV prophylaxis in kidney transplant recipients older than 55 years might be beneficial. This issue needs to be proved further in a prospective way.

The prevalence of the group D+/R+ in our study (92%) was very different form that reported by authors from countries like the United Kingdom (25%), Italy (79%) and the United States (39%) (17).

Although previous studies have shown that the D+/R- serostatus confers a significant risk of CMV disease(1), it is not known if this assumption is also valid for regions with a high CMV seroprevalence such as Costa Rica (8). In the present study we showed that this serostatus is not found in our population. Hence, its possible impact on the risk of developing CMV disease seems to be low. Nevertheless, we were unable to properly quantify the risk of this particular group and further studies are warranted.

It has been suggested that the worst graft and patient survival is observed among the group in which the donor and recipient are both positive (18,19). However, there is still some controversy on this issue, since novel findings have not shown this association (17). Our data did not confirm this relationship either. These discrepancies can be attributable to differences in immunosuppressive strategies between centers, as well as different CMV prophylaxis protocols employed by each transplant unit.

Infection with CMV triggers the risk of acute rejection and chronic nephropathy of the graft. The potential mechanisms include overexpression of the major histocompatibility complex molecules, growth factor, cytokines and an up-regulation of adhesion molecules (20). Although we detected a trend towards a higher percentage of acute rejection among cases (32% versus 16%), it was not statistically significant, perhaps due to a small number of cases.

Regarding chronic graft rejection, we did not note any significant difference between cases and controls either. Indeed, recent studies have failed to identify an effect of early CMV infection on long-term graft loss after adjusting for possible confounders (21). A longer follow up of our data could probably detect any difference in this variable.

Our study did not reveal any specific combination of treatment as a risk factor for developing CMV disease, except for thymoglobulin, when used as induction treatment or as a part of the protocol of acute rejection. This association has been confirmed by previous data (10,11,13).

Of particular interest is the lack of association between mycophenolate mofetil and CMV disease. Although it is a subject of current debate, mycophenolate mofetil has been related to this illness as a consequence of an induced immunosuppressive state characterized by a decreased cellular and humoral response (22,23).Nevertheless, it must be highlighted that this association seems to be related to doses higher than three grams per day, since lower doses are not consistently related to CMV disease as our data shows (24,25).

Finally, we observed an increased incidence of pneumocystis and pulmonary aspergillosis among cases. This observation has been documented in previous reports(26,27) and can be the result of an immunosuppressive state induced by CMV infection. The retrospective nature of our data precluded further investigation of this hypothesis.

## 6. Conclusions

Although our data was drawn from a single-center in a retrospective design, our findings suggest that new risk factors for CMV disease must be considered in a region with high CMV seroprevalence. In conclusion, only recipient age and the use of ATG were independently associated with the risk of CMV disease.

## Limitations of the study

Our research has several limitations due to its retrospective design, which can be a source of selection bias. Besides, the external validity of our findings can be compromised as a consequence of the single-center design of the study.

## Acknowledgements

We thank Dr. Fabian Sanabria for his technical help regarding this study.

## Authors’ contribution

VNR and ARE contributed to this research through the study conception, design and acquisition of data. ARE was in charge of the analysis and interpretation of data. AHM contributed with a critical revision of the paper. AC was on charge of acquiring data as well as the drafting of the manuscript. All the authors read and approved the final version of this paper.

## Conflicts of interest

The authors declare no conflict of interest.

## Funding/Support

This article is extracted from the nephrology thesis of Viviana Navarro-Rodriguez. There has been no significant financial support for this work that could have influenced its outcome.
